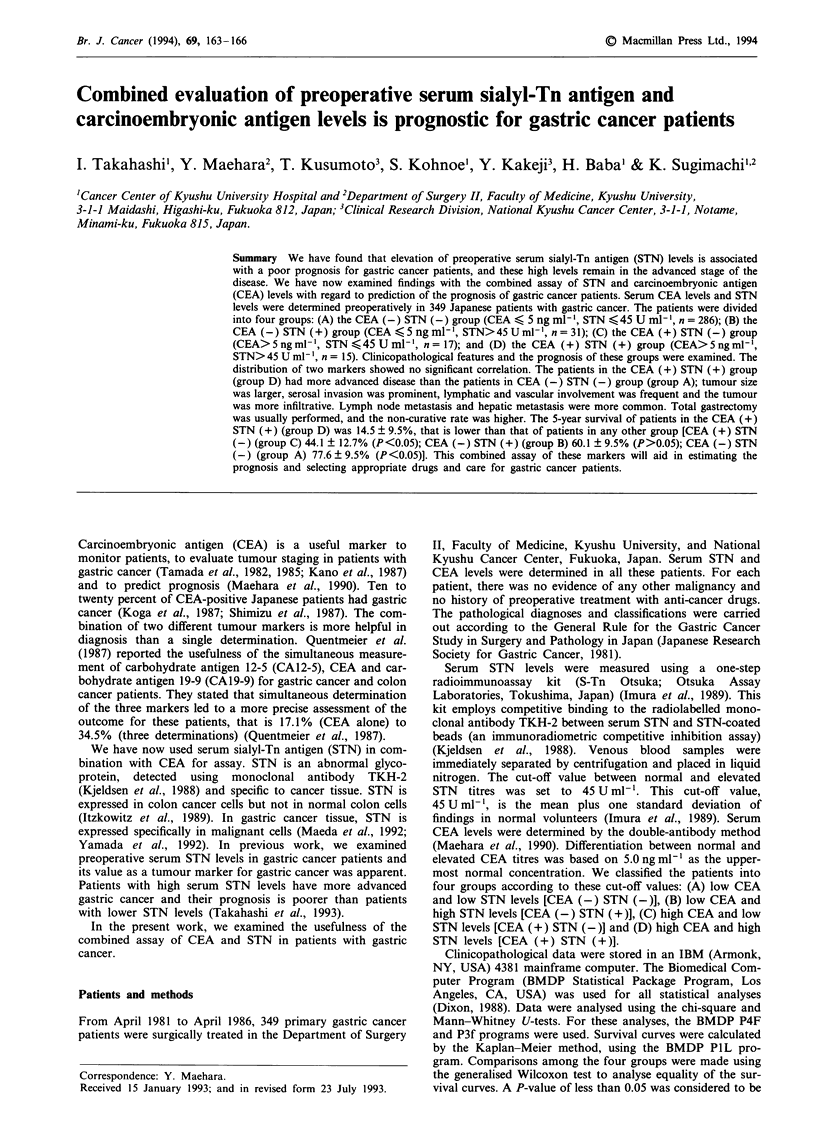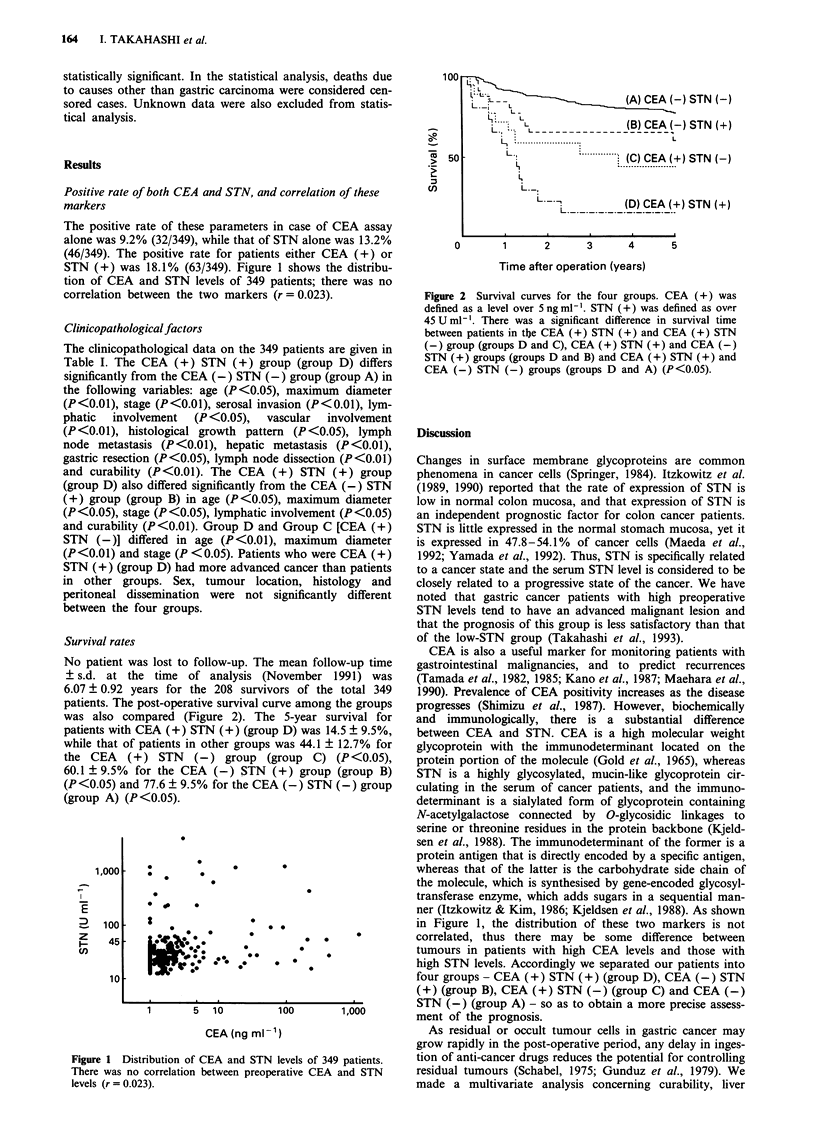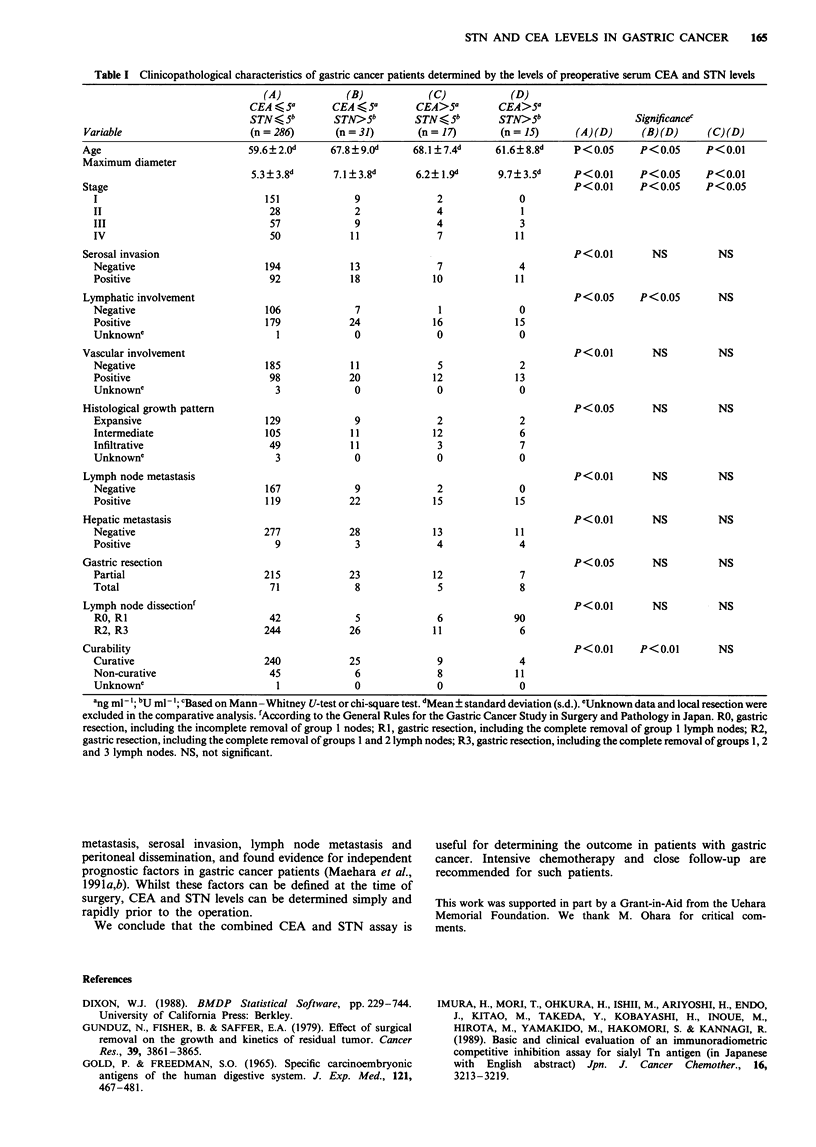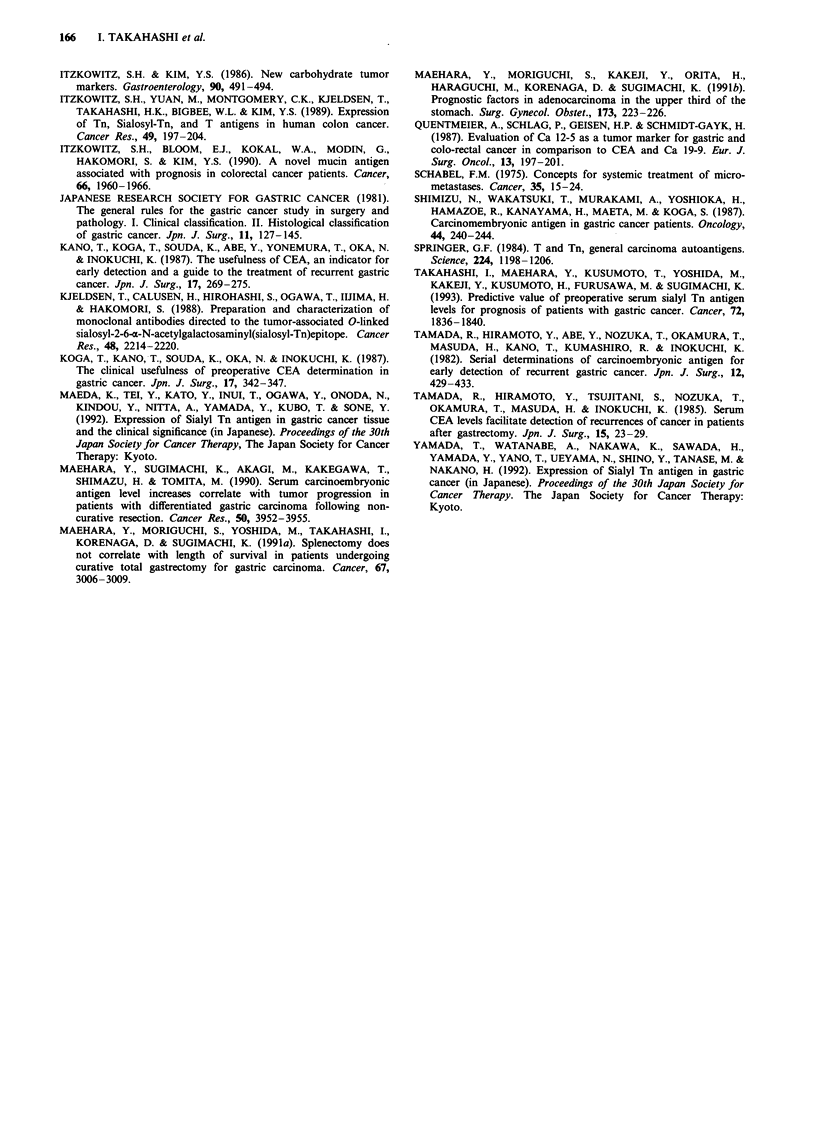# Combined evaluation of preoperative serum sialyl-Tn antigen and carcinoembryonic antigen levels is prognostic for gastric cancer patients.

**DOI:** 10.1038/bjc.1994.27

**Published:** 1994-01

**Authors:** I. Takahashi, Y. Maehara, T. Kusumoto, S. Kohnoe, Y. Kakeji, H. Baba, K. Sugimachi

**Affiliations:** Cancer Center of Kyushu University Hospital, Fukuoka, Japan.

## Abstract

We have found that elevation of preoperative serum sialyl-Tn antigen (STN) levels is associated with a poor prognosis for gastric cancer patients, and these high levels remain in the advanced stage of the disease. We have now examined findings with the combined assay of STN and carcinoembryonic antigen (CEA) levels with regard to prediction of the prognosis of gastric cancer patients. Serum CEA levels and STN levels were determined preoperatively in 349 Japanese patients with gastric cancer. The patients were divided into four groups: (A) the CEA (-) STN (-) group (CEA < or = 5 ng ml-1, STN < or = 45 U ml-1, n = 286); (B) the CEA (-) STN (+) group (CEA < or = 5 ng ml-1, STN > 45 U ml-1, n = 31); (C) the CEA (+) STN (-) group (CEA > 5 ng ml-1, STN < or = 45 U ml-1, n = 17); and (D) the CEA (+) STN (+) group (CEA > 5 ng ml-1, STN > 45 U ml-1, n = 15). Clinicopathological features and the prognosis of these groups were examined. The distribution of two markers showed no significant correlation. The patients in the CEA (+) STN (+) group (group D) had more advanced disease than the patients in CEA (-) STN (-) group (group A); tumour size was larger, serosal invasion was prominent, lymphatic and vascular involvement was frequent and the tumour was more infiltrative. Lymph node metastasis and hepatic metastasis were more common. Total gastrectomy was usually performed, and the non-curative rate was higher. The 5-year survival of patients in the CEA (+) STN (+) (group D) was 14.5 +/- 9.5%, that is lower than that of patients in any other group [CEA (+) STN (-) (group C) 44.1 +/- 12.7% (P < 0.05); CEA (-) STN (+) (group B) 60.1 +/- 9.5% (P > 0.05); CEA (-) STN (-) (group A) 77.6 +/- 9.5% (P < 0.05)]. This combined assay of these markers will aid in estimating the prognosis and selecting appropriate drugs and care for gastric cancer patients.


Br. J. Cancer (1994), 69, 163-166 © Macmillan Press Ltd., 1994

### Combined evaluation of preoperative serum sialyl-Tn antigen and carcinoembryonic antigen levels is prognostic for gastric cancer patients

I. Takahashi[1], Y. Maehara[2], T. Kusumoto[3], S. Kohnoe[1], Y. Kakeji[3], H. Baba[1] & K. Sugimachi[1,2]

[1]Cancer Center of Kyushu University Hospital and [2]Department of Surgery II, Faculty of Medicine, Kyushu University, 3-1-1 Maidashi, Higashi-ku, Fukuoka 812, Japan; [3]Clinical Research Division, National Kyushu Cancer Center, 3-1-1, Notame, Minami-ku, Fukuoka 815, Japan.

**Summary** We have found that elevation of preoperative serum sialyl-Tn antigen (STN) levels is associated with a poor prognosis for gastric cancer patients, and these high levels remain in the advanced stage of the disease. We have now examined findings with the combined assay of STN and carcinoembryonic antigen (CEA) levels with regard to prediction of the prognosis of gastric cancer patients. Serum CEA levels and STN levels were determined preoperatively in 349 Japanese patients with gastric cancer. The patients were divided into four groups: (A) the CEA (−) STN (−) group (CEA $\leqslant$ 5 ng ml$^{-1}$, STN $\leqslant$45 U ml$^{-1}$, $n$ = 286); (B) the CEA (−) STN (+) group (CEA $\leqslant$5 ng ml$^{-1}$, STN>45 U ml$^{-1}$, $n$ = 31); (C) the CEA (+) STN (−) group (CEA>5 ng ml$^{-1}$, STN $\leqslant$45 U ml$^{-1}$, $n$ = 17); and (D) the CEA (+) STN (+) group (CEA>5 ng ml$^{-1}$, STN>45 U ml$^{-1}$, $n$ = 15). Clinicopathological features and the prognosis of these groups were examined. The distribution of two markers showed no significant correlation. The patients in the CEA (+) STN (+) group (group D) had more advanced disease than the patients in CEA (−) STN (−) group (group A); tumour size was larger, serosal invasion was prominent, lymphatic and vascular involvement was frequent and the tumour was more infiltrative. Lymph node metastasis and hepatic metastasis were more common. Total gastrectomy was usually performed, and the non-curative rate was higher. The 5-year survival of patients in the CEA (+) STN (+) (group D) was 14.5 ± 9.5%, that is lower than that of patients in any other group [CEA (+) STN (−) (group C) 44.1 ± 12.7% ($P$<0.05); CEA (−) STN (+) (group B) 60.1 ± 9.5% ($P$>0.05); CEA (−) STN (−) (group A) 77.6 ± 9.5% ($P$<0.05)]. This combined assay of these markers will aid in estimating the prognosis and selecting appropriate drugs and care for gastric cancer patients.

Carcinoembryonic antigen (CEA) is a useful marker to monitor patients, to evaluate tumour staging in patients with gastric cancer (Tamada *et al.*, 1982, 1985; Kano *et al.*, 1987) and to predict prognosis (Maehara *et al.*, 1990). Ten to twenty percent of CEA-positive Japanese patients had gastric cancer (Koga *et al.*, 1987; Shimizu *et al.*, 1987). The combination of two different tumour markers is more helpful in diagnosis than a single determination. Quentmeier *et al.* (1987) reported the usefulness of the simultaneous measurement of carbohydrate antigen 12-5 (CA12-5), CEA and carbohydrate antigen 19-9 (CA19-9) for gastric cancer and colon cancer patients. They stated that simultaneous determination of the three markers led to a more precise assessment of the outcome for these patients, that is 17.1% (CEA alone) to 34.5% (three determinations) (Quentmeier *et al.*, 1987).

We have now used serum sialyl-Tn antigen (STN) in combination with CEA for assay. STN is an abnormal glycoprotein, detected using monoclonal antibody TKH-2 (Kjeldsen *et al.*, 1988) and specific to cancer tissue. STN is expressed in colon cancer cells but not in normal colon cells (Itzkowitz *et al.*, 1989). In gastric cancer tissue, STN is expressed specifically in malignant cells (Maeda *et al.*, 1992; Yamada *et al.*, 1992). In previous work, we examined preoperative serum STN levels in gastric cancer patients and its value as a tumour marker for gastric cancer was apparent. Patients with high serum STN levels have more advanced gastric cancer and their prognosis is poorer than patients with lower STN levels (Takahashi *et al.*, 1993).

In the present work, we examined the usefulness of the combined assay of CEA and STN in patients with gastric cancer.

### Patients and methods

From April 1981 to April 1986, 349 primary gastric cancer patients were surgically treated in the Department of Surgery II, Faculty of Medicine, Kyushu University, and National Kyushu Cancer Center, Fukuoka, Japan. Serum STN and CEA levels were determined in all these patients. For each patient, there was no evidence of any other malignancy and no history of preoperative treatment with anti-cancer drugs. The pathological diagnoses and classifications were carried out according to the General Rule for the Gastric Cancer Study in Surgery and Pathology in Japan (Japanese Research Society for Gastric Cancer, 1981).

Serum STN levels were measured using a one-step radioimmunoassay kit (S-Tn Otsuka; Otsuka Assay Laboratories, Tokushima, Japan) (Imura *et al.*, 1989). This kit employs competitive binding to the radiolabelled monoclonal antibody TKH-2 between serum STN and STN-coated beads (an immunoradiometric competitive inhibition assay) (Kjeldsen *et al.*, 1988). Venous blood samples were immediately separated by centrifugation and placed in liquid nitrogen. The cut-off value between normal and elevated STN titres was set to 45 U ml$^{-1}$. This cut-off value, 45 U ml$^{-1}$, is the mean plus one standard deviation of findings in normal volunteers (Imura *et al.*, 1989). Serum CEA levels were determined by the double-antibody method (Maehara *et al.*, 1990). Differentiation between normal and elevated CEA titres was based on 5.0 ng ml$^{-1}$ as the uppermost normal concentration. We classified the patients into four groups according to these cut-off values: (A) low CEA and low STN levels [CEA (−) STN (−)], (B) low CEA and high STN levels [CEA (−) STN (+)], (C) high CEA and low STN levels [CEA (+) STN (−)] and (D) high CEA and high STN levels [CEA (+) STN (+)].

Clinicopathological data were stored in an IBM (Armonk, NY, USA) 4381 mainframe computer. The Biomedical Computer Program (BMDP Statistical Package Program, Los Angeles, CA, USA) was used for all statistical analyses (Dixon, 1988). Data were analysed using the chi-square and Mann–Whitney $U$-tests. For these analyses, the BMDP P4F and P3f programs were used. Survival curves were calculated by the Kaplan–Meier method, using the BMDP P1L program. Comparisons among the four groups were made using the generalised Wilcoxon test to analyse equality of the survival curves. A $P$-value of less than 0.05 was considered to be

Correspondence: Y. Maehara.
Received 15 January 1993; and in revised form 23 July 1993.

statistically significant. In the statistical analysis, deaths due to causes other than gastric carcinoma were considered censored cases. Unknown data were also excluded from statistical analysis.

### Results

*Positive rate of both CEA and STN, and correlation of these markers*

The positive rate of these parameters in case of CEA assay alone was 9.2% (32/349), while that of STN alone was 13.2% (46/349). The positive rate for patients either CEA (+) or STN (+) was 18.1% (63/349). Figure 1 shows the distribution of CEA and STN levels of 349 patients; there was no correlation between the two markers ($r = 0.023$).

*Clinicopathological factors*

The clinicopathological data on the 349 patients are given in Table I. The CEA (+) STN (+) group (group D) differs significantly from the CEA (−) STN (−) group (group A) in the following variables: age ($P<0.05$), maximum diameter ($P<0.01$), stage ($P<0.01$), serosal invasion ($P<0.01$), lymphatic involvement ($P<0.05$), vascular involvement ($P<0.01$), histological growth pattern ($P<0.05$), lymph node metastasis ($P<0.01$), hepatic metastasis ($P<0.01$), gastric resection ($P<0.05$), lymph node dissection ($P<0.01$) and curability ($P<0.01$). The CEA (+) STN (+) group (group D) also differed significantly from the CEA (−) STN (+) group (group B) in age ($P<0.05$), maximum diameter ($P<0.05$), stage ($P<0.05$), lymphatic involvement ($P<0.05$) and curability ($P<0.01$). Group D and Group C [CEA (+) STN (−)] differed in age ($P<0.01$), maximum diameter ($P<0.01$) and stage ($P<0.05$). Patients who were CEA (+) STN (+) (group D) had more advanced cancer than patients in other groups. Sex, tumour location, histology and peritoneal dissemination were not significantly different between the four groups.

*Survival rates*

No patient was lost to follow-up. The mean follow-up time ± s.d. at the time of analysis (November 1991) was 6.07 ± 0.92 years for the 208 survivors of the total 349 patients. The post-operative survival curve among the groups was also compared (Figure 2). The 5-year survival for patients with CEA (+) STN (+) (group D) was 14.5 ± 9.5%, while that of patients in other groups was 44.1 ± 12.7% for the CEA (+) STN (−) group (group C) ($P<0.05$), 60.1 ± 9.5% for the CEA (−) STN (+) group (group B) ($P<0.05$) and 77.6 ± 9.5% for the CEA (−) STN (−) group (group A) ($P<0.05$).

**Figure 1** Distribution of CEA and STN levels of 349 patients. There was no correlation between preoperative CEA and STN levels ($r = 0.023$).

**Figure 2** Survival curves for the four groups. CEA (+) was defined as a level over 5 ng $ml^{-1}$. STN (+) was defined as over 45 U $ml^{-1}$. There was a significant difference in survival time between patients in the CEA (+) STN (+) and CEA (+) STN (−) group (groups D and C), CEA (+) STN (+) and CEA (−) STN (+) groups (groups D and B) and CEA (+) STN (+) and CEA (−) STN (−) groups (groups D and A) ($P<0.05$).

### Discussion

Changes in surface membrane glycoproteins are common phenomena in cancer cells (Springer, 1984). Itzkowitz *et al.* (1989, 1990) reported that the rate of expression of STN is low in normal colon mucosa, and that expression of STN is an independent prognostic factor for colon cancer patients. STN is little expressed in the normal stomach mucosa, yet it is expressed in 47.8–54.1% of cancer cells (Maeda *et al.*, 1992; Yamada *et al.*, 1992). Thus, STN is specifically related to a cancer state and the serum STN level is considered to be closely related to a progressive state of the cancer. We have noted that gastric cancer patients with high preoperative STN levels tend to have an advanced malignant lesion and that the prognosis of this group is less satisfactory than that of the low-STN group (Takahashi *et al.*, 1993).

CEA is also a useful marker for monitoring patients with gastrointestinal malignancies, and to predict recurrences (Tamada *et al.*, 1982, 1985; Kano *et al.*, 1987; Maehara *et al.*, 1990). Prevalence of CEA positivity increases as the disease progresses (Shimizu *et al.*, 1987). However, biochemically and immunologically, there is a substantial difference between CEA and STN. CEA is a high molecular weight glycoprotein with the immunodeterminant located on the protein portion of the molecule (Gold *et al.*, 1965), whereas STN is a highly glycosylated, mucin-like glycoprotein circulating in the serum of cancer patients, and the immunodeterminant is a sialylated form of glycoprotein containing *N*-acetylgalactose connected by *O*-glycosidic linkages to serine or threonine residues in the protein backbone (Kjeldsen *et al.*, 1988). The immunodeterminant of the former is a protein antigen that is directly encoded by a specific antigen, whereas that of the latter is the carbohydrate side chain of the molecule, which is synthesised by gene-encoded glycosyltransferase enzyme, which adds sugars in a sequential manner (Itzkowitz & Kim, 1986; Kjeldsen *et al.*, 1988). As shown in Figure 1, the distribution of these two markers is not correlated, thus there may be some difference between tumours in patients with high CEA levels and those with high STN levels. Accordingly we separated our patients into four groups – CEA (+) STN (+) (group D), CEA (−) STN (+) (group B), CEA (+) STN (−) (group C) and CEA (−) STN (−) (group A) – so as to obtain a more precise assessment of the prognosis.

As residual or occult tumour cells in gastric cancer may grow rapidly in the post-operative period, any delay in ingestion of anti-cancer drugs reduces the potential for controlling residual tumours (Schabel, 1975; Gunduz *et al.*, 1979). We made a multivariate analysis concerning curability, liver

**Table I** Clinicopathological characteristics of gastric cancer patients determined by the levels of preoperative serum CEA and STN levels

| *Variable* | *(A)* *CEA* ⩽ *5*[a] *STN* ⩽ *5*[b] (n = *286*) | *(B)* *CEA* ⩽ *5*[a] *STN* > *5*[b] (n = *31*) | *(C)* *CEA* > *5*[a] *STN* ⩽ *5*[b] (n = *17*) | *(D)* *CEA* > *5*[a] *STN* > *5*[b] (n = *15*) | *Significance*[c] *(A)(D)* | *(B)(D)* | *(C)(D)* |
|---|---|---|---|---|---|---|---|
| Age | 59.6±2.0[d] | 67.8±9.0[d] | 68.1±7.4[d] | 61.6±8.8[d] | P<0.05 | $P<0.05$ | $P<0.01$ |
| Maximum diameter | 5.3±3.8[d] | 7.1±3.8[d] | 6.2±1.9[d] | 9.7±3.5[d] | $P<0.01$ | $P<0.05$ | $P<0.01$ |
| Stage | | | | | $P<0.01$ | $P<0.05$ | $P<0.05$ |
| I | 151 | 9 | 2 | 0 | | | |
| II | 28 | 2 | 4 | 1 | | | |
| III | 57 | 9 | 4 | 3 | | | |
| IV | 50 | 11 | 7 | 11 | | | |
| Serosal invasion | | | | | $P<0.01$ | NS | NS |
| Negative | 194 | 13 | 7 | 4 | | | |
| Positive | 92 | 18 | 10 | 11 | | | |
| Lymphatic involvement | | | | | $P<0.05$ | $P<0.05$ | NS |
| Negative | 106 | 7 | 1 | 0 | | | |
| Positive | 179 | 24 | 16 | 15 | | | |
| Unknown[e] | 1 | 0 | 0 | 0 | | | |
| Vascular involvement | | | | | $P<0.01$ | NS | NS |
| Negative | 185 | 11 | 5 | 2 | | | |
| Positive | 98 | 20 | 12 | 13 | | | |
| Unknown[e] | 3 | 0 | 0 | 0 | | | |
| Histological growth pattern | | | | | $P<0.05$ | NS | NS |
| Expansive | 129 | 9 | 2 | 2 | | | |
| Intermediate | 105 | 11 | 12 | 6 | | | |
| Infiltrative | 49 | 11 | 3 | 7 | | | |
| Unknown[e] | 3 | 0 | 0 | 0 | | | |
| Lymph node metastasis | | | | | $P<0.01$ | NS | NS |
| Negative | 167 | 9 | 2 | 0 | | | |
| Positive | 119 | 22 | 15 | 15 | | | |
| Hepatic metastasis | | | | | $P<0.01$ | NS | NS |
| Negative | 277 | 28 | 13 | 11 | | | |
| Positive | 9 | 3 | 4 | 4 | | | |
| Gastric resection | | | | | $P<0.05$ | NS | NS |
| Partial | 215 | 23 | 12 | 7 | | | |
| Total | 71 | 8 | 5 | 8 | | | |
| Lymph node dissection[f] | | | | | $P<0.01$ | NS | NS |
| R0, R1 | 42 | 5 | 6 | 90 | | | |
| R2, R3 | 244 | 26 | 11 | 6 | | | |
| Curability | | | | | $P<0.01$ | $P<0.01$ | NS |
| Curative | 240 | 25 | 9 | 4 | | | |
| Non-curative | 45 | 6 | 8 | 11 | | | |
| Unknown[e] | 1 | 0 | 0 | 0 | | | |

[a]ng $ml^{-1}$; [b]U $ml^{-1}$; [c]Based on Mann–Whitney *U*-test or chi-square test. [d]Mean ± standard deviation (s.d.). [e]Unknown data and local resection were excluded in the comparative analysis. [f]According to the General Rules for the Gastric Cancer Study in Surgery and Pathology in Japan. R0, gastric resection, including the incomplete removal of group 1 nodes; R1, gastric resection, including the complete removal of group 1 lymph nodes; R2, gastric resection, including the complete removal of groups 1 and 2 lymph nodes; R3, gastric resection, including the complete removal of groups 1, 2 and 3 lymph nodes. NS, not significant.

metastasis, serosal invasion, lymph node metastasis and peritoneal dissemination, and found evidence for independent prognostic factors in gastric cancer patients (Maehara *et al.*, 1991*a*,*b*). Whilst these factors can be defined at the time of surgery, CEA and STN levels can be determined simply and rapidly prior to the operation.

We conclude that the combined CEA and STN assay is useful for determining the outcome in patients with gastric cancer. Intensive chemotherapy and close follow-up are recommended for such patients.

This work was supported in part by a Grant-in-Aid from the Uehara Memorial Foundation. We thank M. Ohara for critical comments.

### References

DIXON, W.J. (1988). *BMDP Statistical Software*, pp. 229–744. University of California Press: Berkley.

GUNDUZ, N., FISHER, B. & SAFFER, E.A. (1979). Effect of surgical removal on the growth and kinetics of residual tumor. *Cancer Res.*, **39**, 3861–3865.

GOLD, P. & FREEDMAN, S.O. (1965). Specific carcinoembryonic antigens of the human digestive system. *J. Exp. Med.*, **121**, 467–481.

IMURA, H., MORI, T., OHKURA, H., ISHII, M., ARIYOSHI, H., ENDO, J., KITAO, M., TAKEDA, Y., KOBAYASHI, H., INOUE, M., HIROTA, M., YAMAKIDO, M., HAKOMORI, S. & KANNAGI, R. (1989). Basic and clinical evaluation of an immunoradiometric competitive inhibition assay for sialyl Tn antigen (in Japanese with English abstract) *Jpn. J. Cancer Chemother.*, **16**, 3213–3219.

ITZKOWITZ, S.H. & KIM, Y.S. (1986). New carbohydrate tumor markers. *Gastroenterology*, **90**, 491–494.

ITZKOWITZ, S.H., YUAN, M., MONTGOMERY, C.K., KJELDSEN, T., TAKAHASHI, H.K., BIGBEE, W.L. & KIM, Y.S. (1989). Expression of Tn, Sialosyl-Tn, and T antigens in human colon cancer. *Cancer Res.*, **49**, 197–204.

ITZKOWITZ, S.H., BLOOM, E.J., KOKAL, W.A., MODIN, G., HAKOMORI, S. & KIM, Y.S. (1990). A novel mucin antigen associated with prognosis in colorectal cancer patients. *Cancer*, **66**, 1960–1966.

JAPANESE RESEARCH SOCIETY FOR GASTRIC CANCER (1981). The general rules for the gastric cancer study in surgery and pathology. I. Clinical classification. II. Histological classification of gastric cancer. *Jpn. J. Surg.*, **11**, 127–145.

KANO, T., KOGA, T., SOUDA, K., ABE, Y., YONEMURA, T., OKA, N. & INOKUCHI, K. (1987). The usefulness of CEA, an indicator for early detection and a guide to the treatment of recurrent gastric cancer. *Jpn. J. Surg.*, **17**, 269–275.

KJELDSEN, T., CALUSEN, H., HIROHASHI, S., OGAWA, T., IIJIMA, H. & HAKOMORI, S. (1988). Preparation and characterization of monoclonal antibodies directed to the tumor-associated *O*-linked sialosyl-2-6-α-N-acetylgalactosaminyl(sialosyl-Tn)epitope. *Cancer Res.*, **48**, 2214–2220.

KOGA, T., KANO, T., SOUDA, K., OKA, N. & INOKUCHI, K. (1987). The clinical usefulness of preoperative CEA determination in gastric cancer. *Jpn. J. Surg.*, **17**, 342–347.

MAEDA, K., TEI, Y., KATO, Y., INUI, T., OGAWA, Y., ONODA, N., KINDOU, Y., NITTA, A., YAMADA, Y., KUBO, T. & SONE, Y. (1992). Expression of Sialyl Tn antigen in gastric cancer tissue and the clinical significance (in Japanese). *Proceedings of the 30th Japan Society for Cancer Therapy*, The Japan Society for Cancer Therapy: Kyoto.

MAEHARA, Y., SUGIMACHI, K., AKAGI, M., KAKEGAWA, T., SHIMAZU, H. & TOMITA, M. (1990). Serum carcinoembryonic antigen level increases correlate with tumor progression in patients with differentiated gastric carcinoma following non-curative resection. *Cancer Res.*, **50**, 3952–3955.

MAEHARA, Y., MORIGUCHI, S., YOSHIDA, M., TAKAHASHI, I., KORENAGA, D. & SUGIMACHI, K. (1991*a*). Splenectomy does not correlate with length of survival in patients undergoing curative total gastrectomy for gastric carcinoma. *Cancer*, **67**, 3006–3009.

MAEHARA, Y., MORIGUCHI, S., KAKEJI, Y., ORITA, H., HARAGUCHI, M., KORENAGA, D. & SUGIMACHI, K. (1991*b*). Prognostic factors in adenocarcinoma in the upper third of the stomach. *Surg. Gynecol. Obstet.*, **173**, 223–226.

QUENTMEIER, A., SCHLAG, P., GEISEN, H.P. & SCHMIDT-GAYK, H. (1987). Evaluation of Ca 12-5 as a tumor marker for gastric and colo-rectal cancer in comparison to CEA and Ca 19-9. *Eur. J. Surg. Oncol.*, **13**, 197–201.

SCHABEL, F.M. (1975). Concepts for systemic treatment of micro-metastases. *Cancer*, **35**, 15–24.

SHIMIZU, N., WAKATSUKI, T., MURAKAMI, A., YOSHIOKA, H., HAMAZOE, R., KANAYAMA, H., MAETA, M. & KOGA, S. (1987). Carcinomembryonic antigen in gastric cancer patients. *Oncology*, **44**, 240–244.

SPRINGER, G.F. (1984). T and Tn, general carcinoma autoantigens. *Science*, **224**, 1198–1206.

TAKAHASHI, I., MAEHARA, Y., KUSUMOTO, T., YOSHIDA, M., KAKEJI, Y., KUSUMOTO, H., FURUSAWA, M. & SUGIMACHI, K. (1993). Predictive value of preoperative serum sialyl Tn antigen levels for prognosis of patients with gastric cancer. *Cancer*, **72**, 1836–1840.

TAMADA, R., HIRAMOTO, Y., ABE, Y., NOZUKA, T., OKAMURA, T., MASUDA, H., KANO, T., KUMASHIRO, R. & INOKUCHI, K. (1982). Serial determinations of carcinoembryonic antigen for early detection of recurrent gastric cancer. *Jpn. J. Surg.*, **12**, 429–433.

TAMADA, R., HIRAMOTO, Y., TSUJITANI, S., NOZUKA, T., OKAMURA, T., MASUDA, H. & INOKUCHI, K. (1985). Serum CEA levels facilitate detection of recurrences of cancer in patients after gastrectomy. *Jpn. J. Surg.*, **15**, 23–29.

YAMADA, T., WATANABE, A., NAKAWA, K., SAWADA, H., YAMADA, Y., YANO, T., UEYAMA, N., SHINO, Y., TANASE, M. & NAKANO, H. (1992). Expression of Sialyl Tn antigen in gastric cancer (in Japanese). *Proceedings of the 30th Japan Society for Cancer Therapy*. The Japan Society for Cancer Therapy: Kyoto.